# Predictive value of liver cirrhosis using metabolite biomarkers of bile acid in the blood: A protocol for systematic review and meta-analysis

**DOI:** 10.1097/MD.0000000000028529

**Published:** 2022-01-28

**Authors:** Xu Han, Juan Wang, Hao Gu, Xing Liao, Miao Jiang

**Affiliations:** Institute of Basic Research in Clinical Medicine, China Academy of Chinese Medical Sciences, Beijing, China.

**Keywords:** Bile acids, Liver cirrhosis, Metabolomics

## Abstract

**Background:**

Previous studies have indicated that the changes of bile acids are associated with liver cirrhosis. The objective of our study is to perform a systematic review to explore the relationship between bile acids and the pathologic process of cirrhosis, and to find minimally invasive, accurate and reliable potential biomarkers for predicting cirrhosis.

**Methods:**

EMBASE, the Cochrane Library, PubMed, Web of Science, WanFang Data and Chinese National Knowledge Infrastructure (CNKI) will be searched, using the search strategy of liver cirrhosis, bile acids and metabolomic. The screening process will be conducted strictly based on inclusion and exclusion criteria. Clinical studies based on human including randomized controlled trial, cohort study and case control study will be included without restriction of time. Cochrane collaboration's tool for assessing risk of bias and Newcastle-Ottawa Scale (NOS) will be applied to assess the risk of bias to randomized controlled trial and observational study, respectively. The bile acids and their concentrate which are different between liver cirrhosis and control group will be the mainly outcome. A qualitative analysis will be performed to profile the trajectory change of bile acids, then the meta-analysis will be done for quantitative analysis.

**Results:**

The bile acids profile of liver cirrhosis that has potential predictive value for cirrhosis will be identified.

**Conclusion:**

The conclusion of this systematic review will finding potential biomarkers for predicting cirrhosis.

**Ethics and dissemination:**

This systematic review is based on published researches, so there is no ethical approval required. We intend to disseminate our findings in a peer-reviewed journal.

## 1 Introduction

Cirrhosis is necrotic inflammation and fibrosis due to various mechanisms of liver injure^[1]^ with the mortality rates ranges from 1% to 67% worldwide.^[2–4]^ The prevalence of cirrhosis and chronic liver disease can be estimated to be 1.5 billion per 100,000 population according to the global burden of disease.^[5]^ However, the exact prevalence rate is difficult to assess, especially in the area restricted by resource,^[6]^ which due to most chronic liver diseases are asymptomatic in the early stage and hard to be detected until clinical decompensation occurs.^[1]^ Current diagnostic methods, including ultrasound, CT, and MRI, show low accuracy in early stages.^[1]^ Liver biopsy, the golden standard for diagnoses, is limited by inherent deficiencies, such as invalidity sampling error, and intra-observer and inter-observer variability.^[7]^ In the early stage of cirrhosis, the disease can be stabilized by abstention from alcohol or an adequate diet. However, as the disease progresses, the treatment effect is not satisfactory and there are no anti-fibrotic drugs authorized by Food and Drug Administration.^[8]^ The only option is a liver transplant in advanced cirrhosis with a low successful rate and huge cost. Therefore, the exploration of predictive biomarkers with high accuracy and minimal invasion for early diagnosis of cirrhosis is urgent and necessary.

Bile acids are a group of chemically similar molecules synthesized in the liver with diverse physical and biologic properties, such as facilitating the emulsion and absorption of dietary fats and lipid-soluble vitamins, regulating pancreatic enzyme secretion and cholecystokinin release.^[9]^ In recent years, bile acids have attracted increasing attention in multiple research fields as signaling molecules to impact various receptors, such as farnesoid X receptor, a specific nuclear receptor, and G-protein coupled bile salt receptor.^[10]^ The activation of these receptors alters gene expression in multiple tissues, leading to changes in not only bile acid metabolism but also glucose homeostasis, lipid and lipoprotein metabolism, energy expenditure, intestinal motility, bacterial growth, inflammation, and the liver-gut axis.^[10,11]^ Therefore, the potential of bile acids as biomarkers for predicting the diseases is highly expected, especially for hepatobiliary diseases and endocrine diseases.^[12–14]^ Yet, due to the numerous quantities, complex chemical structure, and the complication in the biological regulation and physiological functions, we need a powerful platform that can determine bile acid spectrum as comprehensively and accurately as possible to realize their biomarker potential.

As a promising technique using spectrometric and separation techniques, metabolomics can measure a group of small molecular weight metabolites in biological fluids, tissues, cells, and other samples,^[15]^ which can reflect the overall metabolic state of the body in a certain period precisely, even can predict the state of the body after a period of time. Metabolomics has been successfully applied in the field of biomarkers discovery for various diseases, including liver cancer, sepsis, and osteoarthritis.^[7,16,17]^ For example, the predictive value of several blood amino acids for prediabetes and diabetes by metabolomics have been proved.^[13]^ By using metabolomics approach, pancreatic cancer and pancreatitis could be distinguished.^[18]^ Based on recent studies, the metabolic mechanisms for the pathological process of cirrhosis and the disease stage were elucidated by using this new technology.^[12]^

Using the metabolomic technique, the changes of bile acids have been found in patients with liver cirrhosis compared with control group.^[19]^ Primary bile acids were elevated and secondary bile acids were reduced in patients with advanced cirrhosis compared to those with early stage of cirrhosis, and secondary bile acids were detected in all healthy controls, but not necessarily in patients with cirrhosis in feces and blood.^[20]^ The levels of glycerol phosphoserine and taurocholic acid (TCA) were increased, and lysophosphatidylcholine, glycerol phosphocholine, and other metabolites were decreased when comparing hepatitis B cirrhosis group with control group.^[21]^ Bile acids and carnitine were mentioned in a former study to be the promising biomarkers for primary biliary cirrhosis in urine and serum.^[22]^ Some species of bile acids were explored to be capable to distinguish cirrhosis,^[23]^ which the levels between cirrhosis and healthy were tested by different studies.^[19,24,25]^

However, there are still lacking of consistent conclusions in the predictive biomarkers for cirrhosis. Systematic researches are warranted to provide a solid and comprehensive conclusion for optimized clinical strategy. In this systematic review and meta-analysis, we aim to explore the relationship between bile acids and the pathologic process of cirrhosis through the metabolomics approach, and try to find minimally-invasive, accurate and reliable potential biomarkers for predicting cirrhosis based on published literature.

## 2 Methods and analysis

The performing and writing of our systematic review will be guided by the PRISMA 2020 statement.^[26]^ We registered this protocol in the International Prospective Register of Systematic Reviews (PROSPERO) database, number CRD42021238193.

### 2.1 Review question

Through reviewing the published studies, we aim to know whether bile acids is associated with cirrhosis, further exploring the predictive value of bile acids for liver cirrhosis, and try to find the potential biomarkers.

### 2.2 Eligibility criteria

The studies will be included: adult patients with a confirmed clinical diagnosis of liver cirrhosis; the metabolomic profile of bile acids in blood sample being measured and analyzed using metabolomic technique; healthy people serving as controls whose metabolomic profile of bile acids being measured using the same way as patients with cirrhosis; clinical studies based on human including randomized controlled trial, cohort study and case control study without restriction of time, however languages are English and Chinese. Studies will be excluded if: the pregnancy; cirrhosis patient with other liver disease including hepatic encephalopathy, hepatocellular carcinoma; detection during treatment by ursodesoxycholic acid; duplicate data, for example, studies using the same cohort of patients, or published in the both conference and scientific journals, we will compare studies details then only consider the most complete information; the full text is unavailable.

### 2.3 Search strategy

Literature will be searched through the following databases: EMBASE, the Cochrane Library, PubMed, Web of Science, WanFang Data, and Chinese National Knowledge Infrastructure, using the search strategy as MeSH terms, entry terms and keywords of liver cirrhosis, bile acids, and metabolomic. The specific search strategy is available as a pdf attachment, Supplemental Digital Content. We will restrict the results to human studies that were published in English and Chinese. No time restriction will be applied. To include the latest study, we will retrieve it again before we synthesize data.

### 2.4 Data collection and management

**Figure 1:**
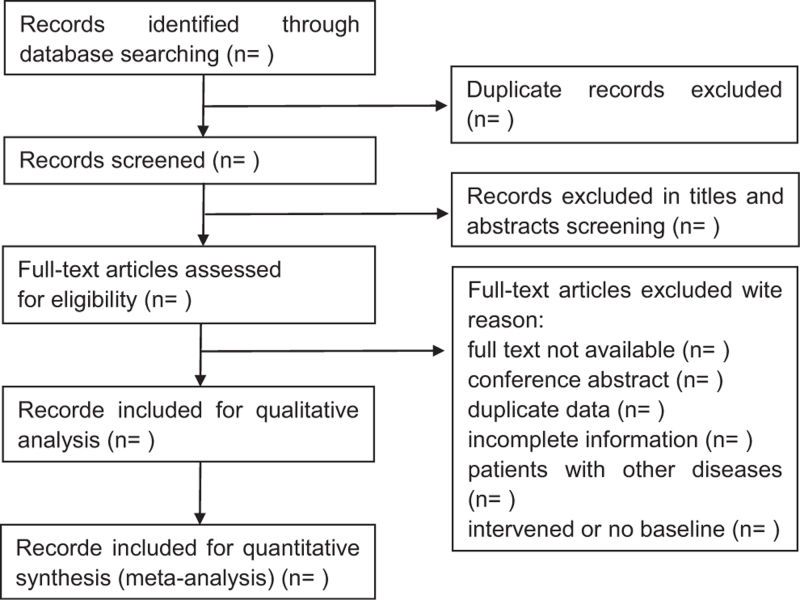
Flow diagram for the assessment of studies identified in the systematic review.

Endnote will be applied to manage the records. The same studies will be deleted. There will be 2 independent reviewers (Han and Wang) to screen the records according to the titles and abstracts. Next, the reserved records will be screened for the full texts. Disagreements will be dealt with by a senior researcher (Jiang) to reach a consensus. Another senior researcher (Liao) will supervise the whole progress and give guidance and help. The flow chart is shown in the Figure 1.

### 2.5 Data extraction

We will extract data including 2 parts as the following items using Excel: the details of studies: the name of the first author; the year of publication; country of origin; study design; language; original inclusion criteria (especially the setting of the participants); sample size (Cirrhosis n = M/F; Health Control n = M/F); age; disease staging, if any; etiology of cirrhosis; the liver biopsy, if any; whether an independent validation cohort is used; and metabolomics technique used to test samples. The results of studies: the bile acids metabolites which are different between groups compared with their variation trend and concentration. Other information of the metabolites such as fold change, area under the receiver operating characteristic curve will be extracted from the passages if available. Contacting the author for raw data will be done when the data is not shown in the passage.

### 2.6 Risk of bias assessment in the included studies

Cochrane collaboration's tool for assessing risk of bias ^[27]^ and Newcastle-Ottawa Scale (http://www.ohri.ca/programs/clinical_epidemiology/oxford.asp) will be applied to assess the risk of bias to randomized controlled trial and observational study, respectively. Cochrane collaboration's tool for assessing risk of bias is specifically aimed at randomized trial, consisting of 6 parts including selection bias, performance bias, detection bias, attrition bias, reporting bias, and other bias.^[27]^ Observational study assessed by Newcastle-Ottawa Scale that the value ≥7 is generally considered as high quality. There will be 2 researchers independently assessing the quality of studies with a group discussion for any divergences.

### 2.7 Data synthesis

The differential metabolites and their changing trend between cirrhosis and healthy control will be recorded, then counting the frequency of their presence for a qualitative analysis.

After qualitative analysis, the concentration of differential metabolites will be a primary outcome to perform a meta-analysis. We will report outcomes as mean difference and standard deviation for continuous outcomes. If there are other parameters like area under the receiver operating characteristic curve, and fold change that are eligible to quantitative synthesis, they will be the second outcomes. The random-effects model will be applied for the possible of huge heterogeneity caused by the different detection methods. *I*^2^ statistic will be used to measure study heterogeneity. If result of *I*^2^ is >50%, it represents substantial heterogeneity, and sensitivity analysis will be performed. Hepatitis B cirrhosis, alcoholic liver cirrhosis and other etiology will be performed by subgroup analysis. Funnel plots will be used to assess publication bias. All the data synthesis will be performed by R software (Version 3.6.2) with meta package. In addition, if there is not enough data to perform a meta-analysis, we will perform a synthesis and report the detail according to the Synthesis Without Meta-analysis (SWiM) guideline.^[28]^

## 3 Discussion

The advantage of metabolomics is easy to collect minimally invasive samples that can be obtained multiple times over a period of time, meanwhile obtaining a large amount of information from the samples.^[29]^ On the other hand, the characteristic of metabolomics is accuracy, speediness, and high flux, so it is also perfect for testing bile acids whose number have found more than 100.^[30–32]^

The previous articles have illustrated that the stages of fibrosis obviously related to the levels of serum total bile acids, primary bile acids, taurochenodeoxycholic acid, glycochenodeoxycholic acid, glycocholic acid, and TCA.^[33]^ They also satisfactorily separate controls from patients with nonalcoholic fatty liver disease cirrhosis.^[34]^ Glycocholic acid, glycochenodeoxycholic acid, TCA, taurochenodeoxycholic acid, and glycoursodeoxycholic acid are changed in hepatitis B-induced cirrhotic patients.^[35]^ TCA is the main elevated bile acid, which can be used as a new target for the prevention and treatment of cirrhosis.^[36]^ It is necessary to explore more specific bile acids species that could become the new targets to prevent and treat cirrhosis, and discover the metabolic mechanism and the internal connection in the future. Therefore, our study, a systematic review about the relationship between bile acids and liver cirrhosis, aims to discover the biomarker to predict cirrhosis.

### 3.1 Strengths and limitations of this study

- A systematic review about the relationship between bile acids and liver cirrhosis based on metabolomics, further exploring the predictive value of bile acids for liver cirrhosis.
- The different etiologies of cirrhosis will be analyzed and assessed, respectively.
- Our study has a meticulous plan, and the whole process is standardized and strict following relevant guidelines for systematic reviews and meta-analysis.
- Restricted by the included researches, we perhaps could not perform a meta-analysis, or the result of synthesis is high heterogeneity, which leads to reduce the quality of evidence. However, we will decrease limitations through effective means to draw a more reliable conclusion based on the available studies.

## Author contributions

XH conceived and design the idea. XH and JW drafted the manuscript, and will independently screen the potential studies, extract data from included studies and assess the risk of bias. HG, MJ, and XL revised the protocol. All authors read and approved the final manuscript.

**Conceptualization:** Xu Han.

**Methodology:** Xing Liao.

**Supervision:** Hao Gu, Xing Liao, Miao Jiang.

**Writing – original draft:** Xu Han, Juan Wang.

## Supplementary Material

Supplemental Digital Content
