# Supplementary material for: Predictive value of liver cirrhosis using metabolite biomarkers of bile acid in the blood: A protocol for systematic review and meta-analysis
Source: Medicine (Baltimore). 2022 Jan 28;101(4):e28529. doi: 10.1097/MD.0000000000028529 (PMC8797474; doi:10.1097/MD.0000000000028529)
Supplement: Supplemental Digital Content [file medi-101-e28529-s001.pdf]

Search strategy

Database	Search number	Query
PubMed	#1	“Bile Acids and Salts” [Mesh]
	#2	((((((Bile Acids[Title/Abstract]) OR (Acids, Bile[Title/Abstract])) OR (Bile Acid[Title/Abstract])) OR (Acid, Bile[Title/Abstract])) OR (Bile Salts[Title/Abstract])) OR (Salts, Bile[Title/Abstract])) OR (Bile Salt[Title/Abstract])) OR (Salt, Bile[Title/Abstract])
	#3	(TBA[Title/Abstract] OR total bile[Title/Abstract] OR CA[Title/Abstract] OR cholic[Title/Abstract] OR DCA[Title/Abstract] OR deox*cholic[Title/Abstract] OR CDCA[Title/Abstract] OR chenodeox*cholic[Title/Abstract] OR UDCA[Title/Abstract] OR ursodeox*cholic[Title/Abstract] OR HDCA[Title/Abstract] OR hyodeoxycholic[Title/Abstract] OR DHCA[Title/Abstract] OR dehydrocholic[Title/Abstract] OR LCA[Title/Abstract] OR lithocholic[Title/Abstract] OR GCA[Title/Abstract] OR glycocholic[Title/Abstract] OR GDCA[Title/Abstract] OR glycodeoxycholic[Title/Abstract] OR GCDCA[Title/Abstract] OR glycochenodeoxycholic[Title/Abstract] OR GUDCA[Title/Abstract] OR glycoursodeoxycholic[Title/Abstract] OR GHDCA[Title/Abstract] OR glycohyodeoxycholic[Title/Abstract] OR GLCA[Title/Abstract] OR glycolithocholic[Title/Abstract] OR TCA[Title/Abstract] OR taurocholic[Title/Abstract] OR TDCA[Title/Abstract] OR taurodeoxycholic[Title/Abstract] OR TCDCA[Title/Abstract] OR taurochenodeoxycholic[Title/Abstract] OR TUDCA[Title/Abstract] OR tauroursodeoxycholic[Title/Abstract]) AND (acid*[Title/Abstract])
	#4	#1 or #2 or #3
	#5	“Liver Cirrhosis” [Mesh]
	#6	((((Hepatic Cirrhosis[Title/Abstract]) OR (Cirrhosis, Hepatic[Title/Abstract])) OR (Cirrhosis, Liver[Title/Abstract])) OR (Fibrosis, Liver[Title/Abstract])) OR (Liver Fibrosis[Title/Abstract])
	#7	(((((((cirrho*[Title/Abstract]) OR (cirrhotic[Title/Abstract])) OR (cirrhotics[Title/Abstract])) OR (liver cirrhosis, alcoholic[Title/Abstract])) OR (liver cirrhosis, biliary[Title/Abstract])) OR (viral cirrhosis[Title/Abstract])) OR (hepatitis B virus-related cirrhosis[Title/Abstract])) OR (cryptogenic cirrhosis[Title/Abstract])
	#8	#5 or #6 or #7
	#9	“Metabolomics” [Mesh]
	#10	((Metabolomic[Title/Abstract]) OR

		(Metabonomics[Title/Abstract])) OR (Metabonomic[Title/Abstract])
	#11	(((((((((((((((((((((((((Metabolomic*[Title/Abstract]) OR (Metabonomic*[Title/Abstract])) OR (Metabolit*[Title/Abstract])) OR (H NMR[Title/Abstract])) OR (nuclear magnetic resonance spectroscopy[Title/Abstract])) OR (proton NMR[Title/Abstract])) OR (proton nuclear magnetic resonance[Title/Abstract])) OR (gas hromatogram*[Title/Abstract])) OR (GC-MS[Title/Abstract])) OR (gas chromatograph-mass spectrometry[Title/Abstract])) OR (GC-TOF-MS[Title/Abstract])) OR (gas chromatography/time-of-flight mass spectrometry[Title/Abstract])) OR (liquid hromatogram*[Title/Abstract])) OR (LC-MS[Title/Abstract])) OR (liquid-chromatography mass spectrometry[Title/Abstract])) OR (TQ MS[Title/Abstract])) OR (triple quadrupole mass spectrometry[Title/Abstract])) OR (UPLC[Title/Abstract])) OR (ultra performance liquid chromatograph*[Title/Abstract])) OR (ultra-performance liquid chromatograph*[Title/Abstract])) OR (HPLC[Title/Abstract])) OR (high performance liquid chrormatograph*[Title/Abstract])) OR (high-performance liquid chrormatograph*[Title/Abstract])) OR (UHPLC-MS/MS[Title/Abstract])) OR (ultra-high performance liquid chromatography/tandem mass spectrometry[Title/Abstract])) OR (UPLC-QTOF-MS[Title/Abstract])) OR (ultraperformance liquid chromatography quadruple time-of-flight mass spectrometer[Title/Abstract])) OR (UHPLC-TQ-MS[Title/Abstract])) OR (ultra-high performance liquid chromatography triple quadrupole mass spectrometry[Title/Abstract])
	#12	#9 or #10 or #11
	#13	#4 and #8 and #12
	#14	“Animals”[Mesh]
	#15	“Humans”[Mesh]
	#16	(“Animals”[Mesh]) NOT (“Humans”[Mesh])
	#17	#13 not #16
